# Rapid, sensitive, and highly specific diagnosis of respiratory syncytial virus using recombinase polymerase amplification-based biosensor and fluorescence detection

**DOI:** 10.3389/fcimb.2026.1838582

**Published:** 2026-07-01

**Authors:** Lei Yu, Bo Peng, Qingjing Du, Yangyang Wu, Shuang Liu, Chunmei Zhu, Chuanhe Liu

**Affiliations:** 1 Capital Institute of Pediatrics, Chinese Academy of Medical Sciences & Peking Union Medical College, Beijing, China; 2 Capital Center for Children’s Health, Capital Medical University, Capital Institute of Pediatrics, Beijing, China

**Keywords:** biosensor, fluorescence detection, point-of-care, recombinase polymerase amplification, respiratory syncytial virus

## Abstract

Respiratory syncytial virus (RSV) is a major cause of acute lower respiratory tract infections in children, highlighting the need for rapid and accessible molecular detection. Here, we developed two recombinase polymerase amplification (RPA)-based assays for the detection and differentiation of RSV-A and RSV-B, combining fluorescence readout or lateral flow biosensor (LFB) visualization, termed RSV-RPA-FLU and RSV-RPA-LFB, respectively. The workflow can be completed within 40 min, including sample preparation for 15 min, isothermal amplification for 20 min, and result interpretation within 5 min. Analytical sensitivity testing showed that the RSV-RPA-FLU assay detected both RSV-A and RSV-B at 10^4^ copies/μL. The RSV-RPA-LFB assay showed higher sensitivity, with detection limits of 6.05 × 10³ copies/μL for RSV-A and 7.80 × 10² copies/μL for RSV-B. Specificity analysis against 11 non-RSV respiratory pathogens showed no cross-reactivity. Preliminary evaluation using simulated clinical samples further supported the feasibility of both assays in complex sample matrices. These results indicate that the RSV-RPA-FLU and RSV-RPA-LFB assays provide rapid, specific, and operationally simple analytical platforms for RSV-A/RSV-B detection and differentiation, holding great potential for point-of-care RSV diagnosis and global prevention and control.

## Background

Respiratory syncytial virus (RSV) is a leading viral cause of acute lower respiratory tract infections (ALRTIs), particularly in pediatric populations, and imposes a substantial burden on global public health (PERCH, 2019). Although RSV infection often presents with nonspecific influenza-like symptoms, such as cough, fever, and wheezing, it is associated with considerable morbidity and mortality related to acute respiratory infections (ARIs) worldwide (Guzman-Holst et al., 2025). RSV is classified into two major antigenic subtypes, RSV-A and RSV-B, which co-circulate during epidemic seasons and recur annually (Tao et al., 2025). These epidemiological and clinical features underscore the urgent need for rapid and accurate diagnostic strategies to support early prevention and control in both household and healthcare settings.

To meet this need, a variety of diagnostic approaches have been developed; however, conventional methods still have important limitations. Viral culture was historically regarded as the reference standard for viral diagnosis, but its labor-intensive procedures, long turnaround time, and high operational cost have limited its routine use and lead to its gradual replacement by faster and more practical methods (Henrickson and Hall, 2007; Onwuchekwa et al., 2023). Antigen-based assays, including immunofluorescence assays and enzyme-linked immunosorbent assays (ELISAs), are widely used because of their rapid reporting and operational simplicity (Popow-Kraupp and Aberle, 2011). However, their analytical sensitivity may be insufficient, particularly in samples with low viral loads. Molecular diagnostic methods, especially polymerase chain reaction (PCR)-based assays, have greatly improved the sensitivity and specificity of pathogen detection and have become widely used for the diagnosis of infectious diseases (Fakruddin et al., 2013). Nevertheless, PCR generally requires thermal cycling instruments, trained personnel, and relatively complex laboratory workflows, which restrict its broader application in point-of-care (POC) and resource-limited settings.

The World Health Organization (WHO) has proposed the ASSURED criteria for POC diagnostics, emphasizing that ideal tests should be affordable, sensitive, specific, user-friendly, rapid and robust, equipment-free or simple to operate, and deliverable to end users (Martzy et al., 2019). In this context, isothermal amplification techniques (IATs) have attracted increasing attention as promising alternatives to conventional PCR. Unlike PCR, which depends on repeated thermal cycling, IATs enable nucleic acid amplification at a constant temperature and can be performed using simple heating devices, such as a water bath or heating block (Zhou et al., 2023; Enyetornye et al., 2025; You et al., 2025). Recent advances in microfluidic biosensing and integrated nucleic acid detection platforms further demonstrate the potential of combining sample processing, isothermal amplification, and signal readout for rapid infectious disease diagnosis (Shen et al., 2023; Xie et al., 2025; Li et al., 2026). For example, microfluidic systems integrating nucleic acid extraction, RPA, and CRISPR-based detection have been reported for respiratory virus or bacterial detection, highlighting the value of compact and integrated platforms for rapid molecular diagnostics (Shen et al., 2023; Xie et al., 2025).

Among IATs, recombinase polymerase amplification (RPA) is particularly attractive because it enables rapid exponential amplification under low and constant temperature conditions without the need for thermal denaturation of the template (Piepenburg et al., 2006). Compared with conventional PCR, which typically requires sophisticated instrumentation and longer reaction times, RPA is simpler, faster, and more compatible with decentralized testing. In parallel, lateral flow biosensors (LFBs) provide a rapid, cost-effective, and instrument-free readout format, making them suitable for POC and field applications, especially in resource-limited areas (Singh et al., 2004; Huang et al., 2019). Advances in microfluidic chip design, micromixing, and particle manipulation have also provided useful engineering strategies for improving reagent mixing, reaction efficiency, and device integration in miniaturized diagnostic systems (Han et al., 2023, 2024b, a; Han et al., 2025, 2026).

In this study, we developed two RPA-based assays, RSV-RPA-FLU and RSV-RPA-LFB, for the rapid detection and differentiation of RSV-A and RSV-B. The fluorescence-based assay enables real-time monitoring of amplification, whereas the LFB-based assay provides a simple visual readout without the need for complex instrumentation. By integrating rapid isothermal amplification with fluorescence and lateral-flow readout strategies, the proposed assays aim to achieve sensitive, specific, rapid, and portable RSV detection. This approach may provide a practical diagnostic tool for the early detection of pediatric RSV infection, thereby improving clinical decision-making, facilitating timely infection control, and reducing disease transmission.

## Materials and methods

### Experimental instruments and reagents

The following instruments and reagents were used in this study: PCR thermocycler (Bio-Rad Laboratories, Hercules, CA, USA), real-time fluorescence quantitative PCR (qPCR) instrument (7500, Thermo Fisher Scientific, Applied Biosystems, Waltham, MA, USA), NanoDrop microvolume spectrophotometer (NanoDrop 2000, Thermo Fisher Scientific, USA), TwistAmp exo kits and TwistAmp Basic kits (TwistDx Ltd., Cambridge, UK), primers and probes (Beijing SinoGenoMax Co., Ltd., Beijing, China), and gold nanoparticle–based LFB (Huidexin Biotechnology, Tianjin, China).

### Target DNA, primers, and probes

The target fragments for RSV-A and RSV-B were selected from conserved genomic regions reported by Gong et al. (RSV-A, OQ848527, 2731-3132; RSV-B, KY674983, 2700-3100) (Gong et al., 2022). Based on the conserved target fragments, RPA primers were designed to ensure efficient and specific amplification of the corresponding RSV-A or RSV-B sequences. Probes were then designed within the amplified regions according to the TwistDx RPA probe design guidelines. Candidate primers and probes were further evaluated in silico for sequence length, GC content, potential hairpin formation, self-dimer formation, and sequence specificity. GC content, hairpin ΔG, and self-dimer ΔG values were calculated using IDT OligoAnalyzer. For hairpin and self-dimer analyses, the most negative predicted ΔG value was recorded. The results were interpreted using predefined reference criteria, including GC content of 30–70%, hairpin ΔG preferably ≥ −5 kcal·mol^-1^, and self-dimer ΔG preferably ≥ −6 kcal·mol^-1^. Values outside these reference criteria were not used as absolute exclusion criteria but were considered potential risk indicators requiring experimental validation. For modified probes, the nucleotide sequence was used for thermodynamic prediction, while the corresponding modifications were retained in **Supplementary Table S1** to indicate the actual probe structures used in the assays. BLAST analysis was performed to assess sequence matching to the intended target sequences, and BLAST identity and query coverage were recorded, with 100% identity and 100% query coverage considered supportive of target specificity. The specificity of the candidate primers and probes was also assessed by sequence alignment analysis against five representative RSV-A target sequences and five representative RSV-B target sequences using Jalview software (**Supplementary Figure S1**). Sequences of primers and probes and the corresponding in silico analysis results are listed in **Supplementary Table S1**; **Supplementary Figure S1**. All oligonucleotides were purified by HPLC, diluted to a concentration of 10 μM with nuclease-free water, and stored at −20 °C in the dark.

### RSV-RPA-FLU assay and RSV-RPA-LFB assay

**Figure 1:**
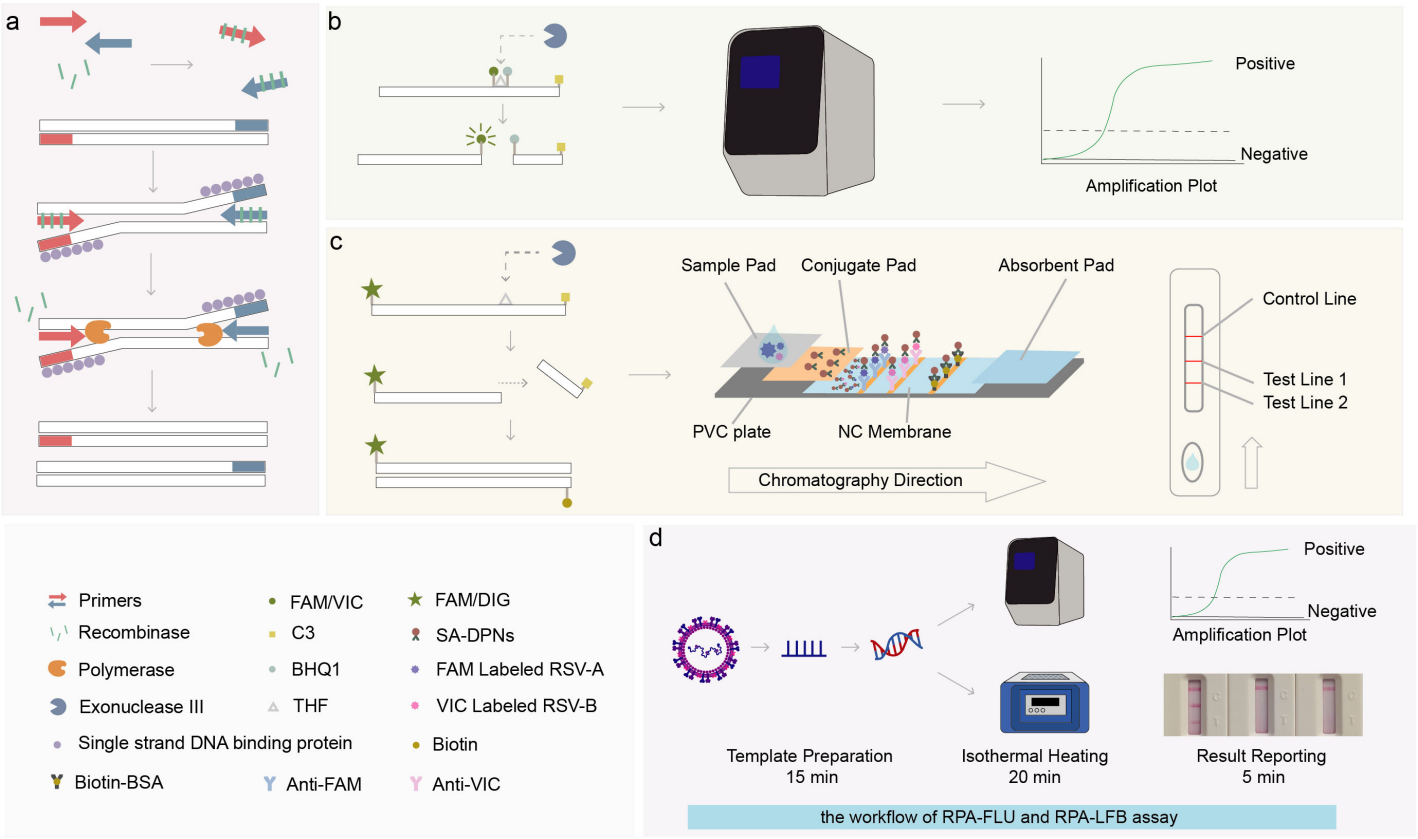
Principles and workflow of RSV-RPA-FLU and RSV-RPA-LFB assays. **(a)** Principle illustration of RPA. **(b)** Principle illustration of RSV-RPA-FLU assay. exo probe: FAM/VIC (fluorophore) and BHQ1 (quencher) flanked tetrahydrofuran (THF) cleavage site and C3-spacer blocked at 3’ end. Target DNA-induced exo probe cleavage releases fluorescence signals and then real-time monitored by real-time PCR instrument. Positive: “S”-shaped curve; Negative: flat baseline. **(c)** Principle illustration of RSV-RPA-LFB assay. nfo probe: 5’ FAM/DIG-labeled, THF located in the middle, 3’ blocked with C3-spacer; reverse primers labeled with biotin. Positive samples show red bands at test line 1 (TL1, RSV-A) and/or 2 (TL2, RSV-B); control line (CL) validates tests. **(d)** Workflow of the RSV-RPA-FLU and RSV-RPA-LFB assays. Both assays were able to complete detection within 40 min, including template preparation for 15 min, isothermal amplification for 20 min, and result reporting for 5 min.

The detection principle of RSV-RPA-FLU and RSV-RPA-LFB assays were shown in **Figure 1** and both assays performed using commercial RPA kits (TwistAmp exo Kit, TwistDx Ltd., UK, catalog number: TAEXO02KIT). RPA master mix was prepared according to the manufacturer’s instructions, and the enzyme and buffer components supplied in the commercial kits were not independently modified. Briefly, the basic RPA reaction mixture contained 1.05 μL each of the standard forward primer and reverse primer (10 μM) (the standard reverse primers were replaced by biotin labeled ones when RSV-RPA-LFB assay performed), 0.3 μL each of probes (exo probes for RSV-RPA-FLU assay, while the nfo probes for RSV-RPA-LFB assay), 29.5 μL of primer-free rehydration buffer, 2 μL of DNA template (2 μL of RSV-A or RSV-B plasmid, or 1 μL each of RSV-A and RSV-B plasmids), and 11.2 μL of nuclease-free water. This mixture was used to resuspend the lyophilized enzyme pellet, and the reaction was initiated by adding 2.5 μL of 280 mM magnesium acetate (MgOAc), followed by incubation at 39 °C for 20 min. Results of RSV-RPA-FLU assay was real-time monitored by real-time fluorescence quantitative PCR (qPCR) instrument and end-point acquired via fluorescence values, while the RSV-RPA-LFB was by visual readout of the LFB, with the co-occurrence of test line1 (TL1) and control line (CL) defined as positive for RSV-A, co-occurrence of test line2 (TL2) and CL defined as positive for RSV-B, while only the CL indicated a negative result. Tests using RSV-A and/or RSV-B plasmid mixture were utilized as positive control, distilled water (DW) as blank control. All experiments were performed in triplicate otherwise stated. The RSV-RPA-FLU and RSV-RPA-LFB assays were used as a qualitative detection method in this study. Although fluorescence signals were monitored in real-time, they were used only to determine positive or negative results, rather than to quantify RSV viral load.

To determine the optimal assay conditions, the concentrations of primers and probes were empirically optimized for each assay. Different primer concentrations (0.11-0.31 μM with an interval of 0.05 for RSV-A, 0.06-0.13 μM with an interval of 0.01 for RSV-B) and probe concentrations (0.06-0.12 μM with an interval of 0.02 for RSV-A, 0.18-0.24 μM with an interval of 0.01 for RSV-B) were evaluated for RSV-RPA-FLU assay. The optimal condition was selected based on rapid fluorescence signal generation, high endpoint fluorescence intensity, and the absence of amplification in the no-template control. For RSV-RPA-LFB assay optimization, different primer (0.03-0.21 μM with an interval of 0.02) and probe concentration (0.04, 0.05, 0.06 μM) and various rations of RSV-A/RSV-B specific components (primer and probe) (3:7, 2:8, 1:9) were assessed. The optimal condition was selected based on a clear and intense test line in positive reactions and the absence of a test line in negative controls.

### Analytical sensitivity evaluation of RSV-RPA-FLU assay and RSV-RPA-LFB assay

The analytical sensitivity of the RSV-RPA-FLU assay and RSV-RPA-LFB assay was evaluated using serial dilutions of two recombinant plasmids containing the respective conserved targets of RSV-A and RSV-B. The initial concentration of each plasmid was determined using a microvolume spectrophotometer, and the plasmid copy number was calculated according to the standard conversion formula for double-stranded DNA mass concentration to molecule copy number as described previously (Hue et al., 2016):


$$\text{Copy number }\left(\text{copies}/\text{μL}\right)\;=\;\left[\text{plasmid concentration }\left(\text{ng}/\text{μL}\right)\;\times \;10^{–9}\;\times \;6.022\;\times \;10^{23}\right]/\left[\text{plasmid length }\left(\text{bp}\right)\;\times \;660\right]$$

where 10^-9^ is the conversion factor from nanograms to grams, 6.022 × 10²³ is Avogadro’s constant, 660 g/mol is the average molecular weight of one base pair of double-stranded DNA, and plasmid length refers to the total length of the recombinant plasmid, including the vector backbone and the inserted target fragment.

The plasmids were 10-fold serially diluted with nuclease-free water to final concentrations ranging from 6.05 × 10^8^ to 6.05 × 10² copies/μL for RSV-A and from 7.80 × 10^8^ to 7.80 × 10² copies/μL for RSV-B. Equal volumes of RSV-A and RSV-B plasmid dilutions at the corresponding dilution level were mixed and then subjected to RSV-RPA-FLU and RSV-RPA-LFB detection in triplicate, with nuclease-free water used as the blank control. In the initial analytical sensitivity screening, each plasmid dilution was tested in triplicate. The putative lowest detectable concentration was defined as the lowest concentration that yielded positive results in all three replicates. To increase the statistical confidence of this preliminary estimate, the putative lowest detectable concentration was subsequently evaluated using 20 independent replicates for each RSV subtype. A tested concentration was considered to meet the detection-limit confirmation criterion when positive results were obtained in at least 95% of replicates, corresponding to at least 19 positive results among 20 replicates. The positivity rate and 95% confidence interval were calculated using the Wilson method.

### Specificity analysis of RSV-RPA-FLU assay and RSV-RPA-LFB assay

To determine the specificity of the assays, a total of 15 samples were tested, including 11 non-RSV pathogens (**Supplementary Table S2**), RSV-A plasmid, RSV-B plasmid, a mixture of both, and DW as blank control. All the pathogens were tested by RSV-RPA-FLU and RSV-RPA-LFB assays under the optimized reaction for three repeats. Results were interpreted as above mentioned.

### Preliminary evaluation using plasmid-spiked nasopharyngeal swab matrix

To preliminarily evaluate the feasibility of the RSV-RPA-FLU and RSV-RPA-LFB assays in a respiratory specimen background, plasmid-spiked nasopharyngeal swab matrix samples were prepared. Briefly, RSV-A and RSV-B plasmid standards were first prepared at different concentrations by serial dilution and then separately mixed with the RSV-negative nasopharyngeal swab matrix to generate RSV-A-spiked (final RSV-A concentration ranging from 6.05×10^8^ copies/μL to 6.05×10^2^ copies/μL) and RSV-B-spiked (final RSV-B concentration ranging from 7.80×10^8^ copies/μL to 7.80×10^2^ copies/μL) matrix samples. Each prepared matrix sample was subjected to nucleic acid extraction and purification using a commercial nucleic acid extraction kit according to the manufacturer’s instructions. The purified nucleic acids were then tested using the RSV-RPA-FLU and RSV-RPA-LFB assays. Each concentration was tested in triplicate. The lowest concentration that yielded positive results in all three replicates was defined as the analytical detection limit under the nasopharyngeal swab matrix condition.

## Result

### Feasibility of the RSV-RPA-FLU and RSV-RPA-LFB assays

Before experimental optimization, the designed primers and probes were evaluated in silico. As shown in **Supplementary Table S1**, the GC contents of all primers and probes ranged from 30.8% to 36.2%. The predicted hairpin ΔG values ranged from -0.38 to -5.22 kcal·mol^-1^, indicating no strong hairpin formation. BLAST analysis showed 100% identity and 100% query coverage for all primers and probes against the intended target sequences. Some probes showed relatively negative self-dimer ΔG values, suggesting potential self-interaction in silico. Therefore, the final suitability of the primer/probe sets was further assessed by experimental validation. During assay optimization and feasibility confirmation, no amplification or false-positive signal was observed in the no-template, blank, or non-target controls, supporting the suitability of the selected primer/probe sets for subsequent RSV-RPA-FLU and RSV-RPA-LFB assays.

After in silico screening, primer and probe concentrations were optimized for both assays before feasibility confirmation. For RSV-RPA-FLU, the optimized conditions were 0.21 μM RSV-A primers (1.05 μL), 0.22 μM RSV-B primers (1.1 μL), 0.10 μM RSV-A exo probe (0.5 μL), and 0.10 μM RSV-B exo probe (0.5 μL), which were determined based on rapid fluorescence signal generation, high endpoint fluorescence intensity, and no amplification in the no-template control (**Supplementary Figure S2**). For RSV-RPA-LFB, the optimal RSV-A/RSV-B component ratio was 1:9, and the final concentrations were 0.02 μM RSV-A primers (0.1 μL), 0.18 μM RSV-B primers (0.9 μL), 0.02 μM RSV-A nfo probe (0.1 μL), and 0.18 μM RSV-B nfo probe (0.9 μL), which determined according to the intensity and clarity of the test line in positive reactions and the absence of test lines in negative controls. The final reaction systems were shown in **Supplementary Table S2** and were used for subsequent sensitivity, specificity, and simulated sample analysis otherwise stated.

**Figure 2:**
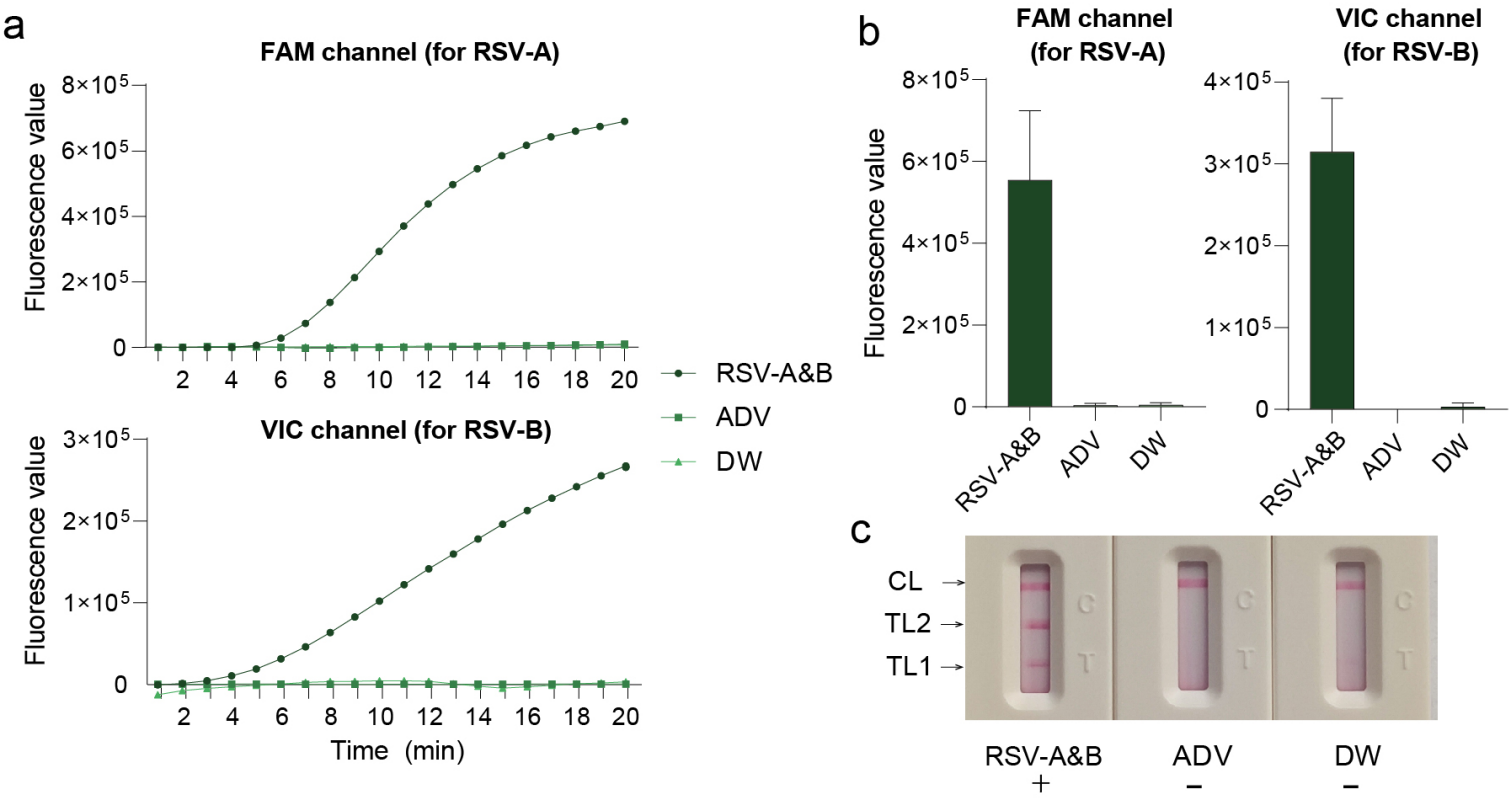
Feasibility confirmation of the RSV-RPA-FLU and RSV-RPA-LFB assays. **(a)** Real-time amplification curve of the RSV-RPA-FLU assay for feasibility confirmation; **(b)** End-point fluorescence value of the RSV-RPA-FLU assay for feasibility confirmation; **(c)** Visual LFB strip readout of the RSV-RPA-LFB for feasibility confirmation. RSV-A and RSV-B plasmid mixture was used as positive control, adenovirus was used as a negative control, and double-distilled water was used as a blank control. In the LFB assay, two visible lines (TL1 and TL2) indicate a positive result, while only the control line (CL) indicates a negative result.

Following optimization, the feasibility of RSV-RPA-FLU and RSV-RPA-LFB assays was confirmed using the mixed RSV-A and RSV-B plasmid solution as positive control, adenovirus as a negative control, and double-distilled water as a blank control. In the RSV-RPA-FLU assay, clear fluorescence amplification curve and increased end-point fluorescence value were observed only in the RSV-A and RSV-B plasmid mixture reaction, whereas no fluorescence amplification was detected in the adenovirus or double-distilled water controls (**Figures 2a, b**; **Supplementary Figure S3**). Consistently, in the RSV-RPA-LFB assay, simultaneous appearance of visible TL1, TL2 and CL presented only in the RSV-A and RSV-B plasmid mixture reaction, while solely appearance of CL was observed from the adenovirus and blank control reactions (**Figure 2c**). These findings indicated that both assays were feasible for detection and differentiation of RSV-A and RSV-B targets.

### Analytical sensitivity of RSV-RPA-FLU assay and RSV-RPA-LFB assay

**Figure 3:**
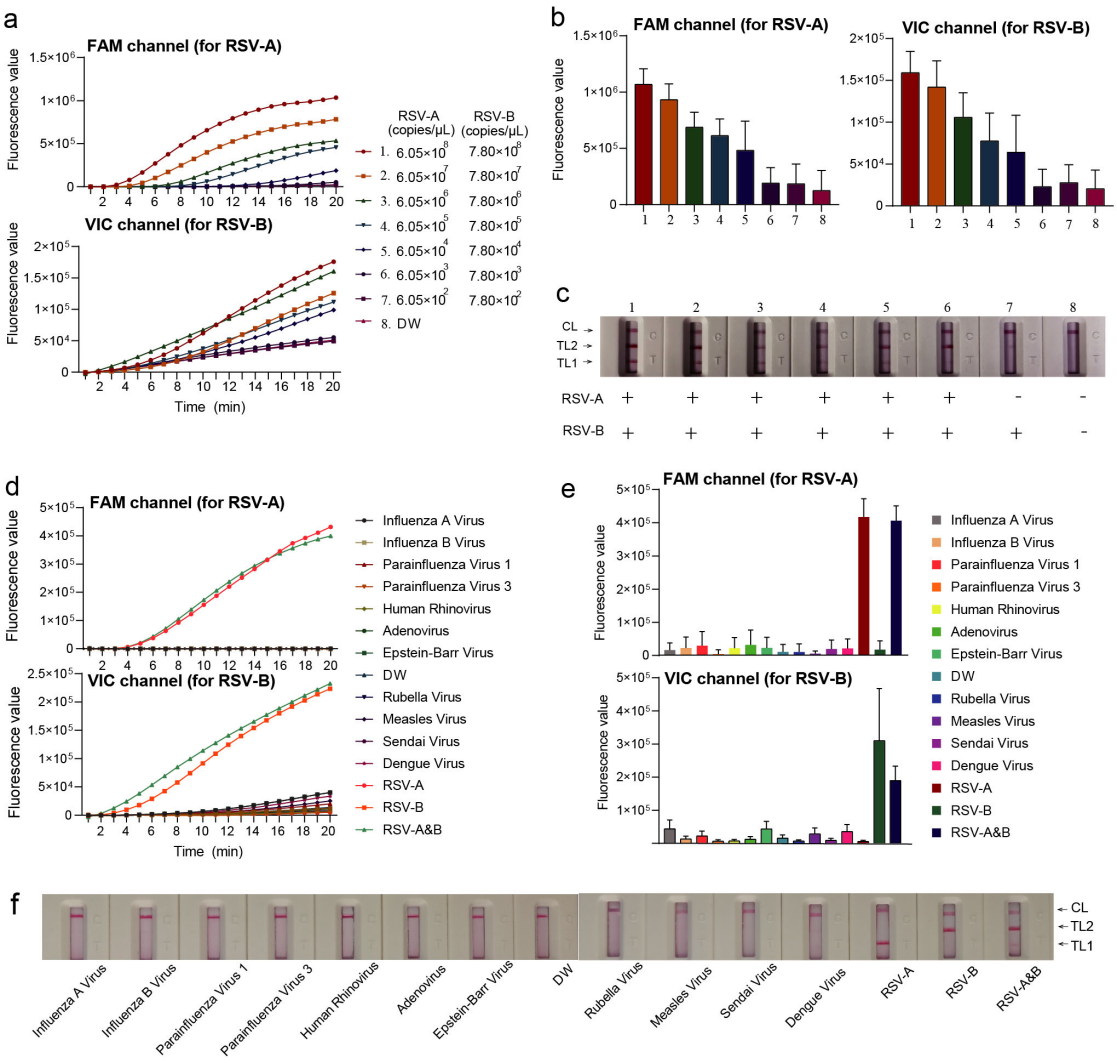
Analytical sensitivity and specificity of the RSV-RPA-FLU and RSV-RPA-LFB assays. **(a)** Real-time amplification curve of serial plasmid dilutions using the RSV-RPA-FLU assay for analytical sensitivity evaluation; **(b)** End-point fluorescence value of serial plasmid dilutions using the RSV-RPA-FLU assay for analytical sensitivity evaluation; **(c)** Visual LFB strip readout of serial plasmid dilutions using RSV-RPA-LFB for analytical sensitivity evaluation. For analytical sensitivity analysis, RSV-A plasmid ranging from 6.05×10^8^ copies/μL to 6.05×10^2^ copies/μL and RSV-B plasmid ranging from 7.80×10^8^ copies/μL to 7.80×10^2^ copies/μL were tested in triplicate. DW was used as blank control. **(d)** Analytical specificity evaluation of the RSV-RPA-FLU assay by real-time fluorescence monitoring; **(e)** Analytical specificity evaluation of the RSV-RPA-FLU assay by end-point fluorescence value; **(f)** Analytical specificity evaluation of the RSV-RPA-LFB by visual LFB strip readout. For analytical specificity analysis, 11 non-RSV pathogens were detected for three times. DW was used as blank control, while RSV-A and/or RSV-B considered as positive controls.

To evaluate the analytical sensitivity of the two methods, serial dilutions of the plasmid mixtures were prepared and detected. Results indicated that both the RSV-RPA-FLU assay and the RSV-RPA-LFB assay were able to stably detect and differentiate RSV-A and RSV-B target sequences (**Figure 3**). Specifically, the lowest plasmid concentration detected by RSV-RPA-FLU assay was 6.05×10^4^ and 7.80×10^4^ copies/μL for RSV-A and RSV-B, respectively (**Figures 3a, b**; **Supplementary Figure S4**), and the RSV-RPA-LFB assay achieve stable detection of RSV-A at a concentration identical to RSV-RPA-FLU assay (6.05×10^3^ copies/μL) and RSV-B at the concentration of 7.80×10^2^ copies/μL (**Figure 3c**). Concentrations lower than these values and the DW were all identified as negative.

To further increase the statistical confidence of the analytical sensitivity assessment, 20 independent replicate reactions were performed at the putative lowest detectable concentrations identified in the initial triplicate screening. For RSV-A, 20 of 20 replicates were positive, corresponding to a positivity rate of 100% with a Wilson 95% confidence interval of 0.839–1.000; for RSV-B, 19 of 20 replicates were positive, corresponding to a positivity rate of 95% with a Wilson 95% confidence interval of approximately 0.764–0.991 (**Supplementary Table S4**). Therefore, both RSV-A and RSV-B met the predefined ≥95% positivity criterion at the tested concentrations. These results confirmed the analytical sensitivity of the RSV-RPA-FLU and RSV-RPA-LFB assays for RSV-A and RSV-B detection with increased statistical confidence.

### Analytical specificity of RSV-RPA-FLU assay and RSV-RPA-LFB assay

The analytical specificity of the two assays was evaluated using 11 non-RSV pathogens that clinically co-circulate with RSV in respiratory infections or were available in our laboratory. RSV-A and/or RSV-B were used as positive controls, and distilled water was used as the negative control. Each non-RSV pathogen was tested in triplicate. As shown in **Figures 3d–f**, **Supplementary Figure S5**, only the positive control reactions produced positive results. All non-RSV pathogens and the distilled water control were negative for RSV-A and RSV-B, with no detectable amplification curve or fluorescence signal observed in the RSV-RPA-FLU assay and only the control line (CL) present on the LFB strips following the RSV-RPA-LFB assay. These results suggest that both assays had good analytical specificity within the tested pathogen panel, with no detectable cross-reactivity observed under the experimental conditions used in this study.

### Preliminary evaluation using plasmid-spiked nasopharyngeal swab matrix

**Figure 4:**
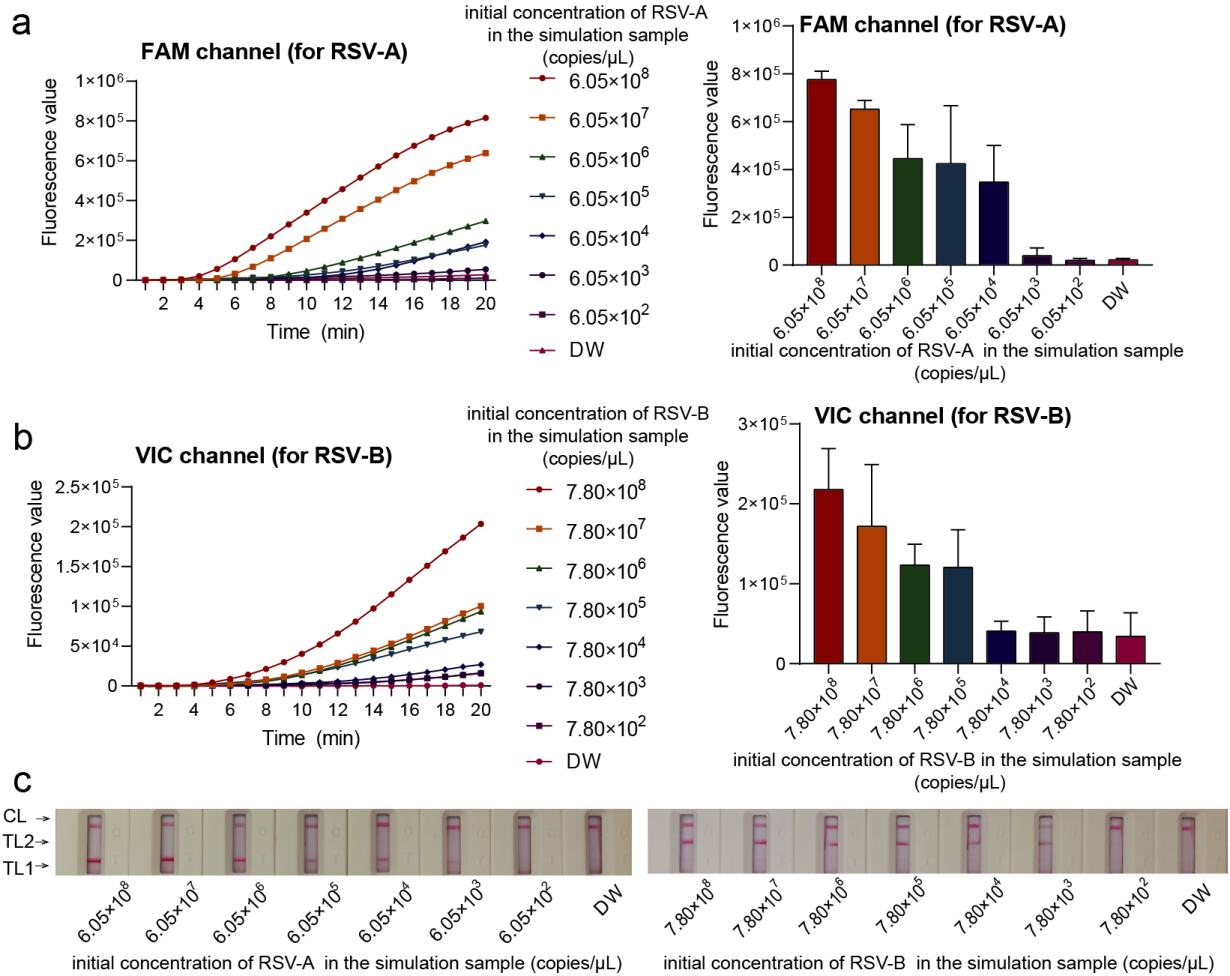
Preliminary evaluation of the RSV-RPA-FLU and RSV-RPA-LFB assays using plasmid-spiked nasopharyngeal swab matrix. **(a)** Real-time amplification curve (left) and end-point fluorescence value (right) of RSV-A detection in the stimulated samples using RSV-RPA-FLU assay for preliminary evaluation; **(b)** Real-time amplification curve (left) and end-point fluorescence value (right) of RSV-B detection in the stimulated samples using RSV-RPA-FLU assay for preliminary evaluation; **(c)** Visual LFB strip readout of RSV-A (left) and RSV-B (right) in the stimulated samples using RSV-RPA-LFB for preliminary evaluation. Concentrations of RSV-A and RSV-B in stimulated nasopharyngeal swab samples ranged from from 6.05×10^8^ copies/μL to 6.05×10^2^ copies/μL and from 7.80×10^8^ copies/μL to 7.80×10^2^ copies/μL, respectively. Each test was performed in triplicate.

Plasmid-spiked nasopharyngeal swab matrix samples were tested in parallel using the RSV-RPA-FLU and RSV-RPA-LFB assays after nucleic acid extraction and purification. For the RSV-RPA-FLU assay, the lowest detectable concentrations were 6.05 × 10^4^ copies/μL for RSV-A and 7.80 × 10^5^ copies/μL for RSV-B, as determined by real-time fluorescence amplification curves and end-point fluorescence values (**Figures 4a, b**; **Supplementary Figure S6**). For the RSV-RPA-LFB assay, both RSV-A and RSV-B were consistently detectable at 10³ copies/μL, as indicated by the presence of visible TL1 or TL2 and CL on the LFB strips (**Figure 4c**). These results suggested that both assays were able to detect RSV in a nasopharyngeal swab matrix background, although this experiment represents a preliminary matrix feasibility assessment rather than clinical validation.

## Discussion

In this study, we developed two RPA-based assays, RSV-RPA-FLU and RSV-RPA-LFB, for the rapid detection and differentiation of RSV-A and RSV-B. Both assays showed favorable analytical sensitivity and specificity using recombinant plasmids and simulated samples, supporting their feasibility as rapid molecular detection platforms.

Compared with conventional RT-qPCR, antigen-based rapid tests, and RPA-CRISPR/Cas12a assays, the RSV-RPA-FLU and RSV-RPA-LFB assays developed in this study show advantages in analytical sensitivity, specificity, simplicity, turnaround time, and equipment requirement. RT-qPCR remains the reference molecular method for RSV diagnosis because of its high diagnostic sensitivity, specificity, and quantitative capability. Its reported detection limits range from several to several hundred RNA copies per reaction, depending on assay design, target region, extraction method, and quantification standard (Wang et al., 2019; Todd et al., 2021). However, their dependence on thermal cycling instruments, trained personnel, laboratory-based workflows and long turn-around time restricted the use in primary-care settings and resource-limited areas. Antigen-based rapid tests are simple, inexpensive, and fast (Chartrand et al., 2015). However, their sensitivity is generally lower than that of nucleic acid amplification methods, especially in samples with low viral burden. RPA-CRISPR/Cas12a assays have also been reported for RSV detection and can achieve high analytical sensitivity (Gong et al., 2022; Hongdan et al., 2024). Nevertheless, these assays usually require RPA amplification, Cas12a-mediated target recognition, and reporter cleavage. This increases reagent complexity and requires stricter control of reaction steps. In comparison, the RSV-RPA-FLU and RSV-RPA-LFB assays developed in this study achieved favorable detection limits of 10^4^ to 10^2^ copies/μL within a short timeframe (~ 40 min, **Figure 1d**) using simple instrument and procedure. The two methods developed in this study generate signals directly through RPA amplification and probe-specific recognition, without an additional CRISPR/Cas-mediated enzymatic step. The fluorescence format enables real-time monitoring or end-point readout, whereas the LFB format allows visual detection without specialized equipment. Additionally, a panel of 11 non-RSV pathogens were detected by the two new methods and no cross-reactivity was observed, indicating their promising potential in detecting and differentiating RSV-A and RSV-B from other closely related pathogens. A detailed comparison of the existing methods in terms of sensitivity, assay time, equipment requirements, relative cost, and application settings is summarized in **Supplementary Table S5**.

In addition to assay sensitivity, specificity and workflow simplicity, the dual-readout design also allows the two formats to be selected according to different testing conditions. The dual-readout design of the RSV-RPA-FLU and RSV-RPA-LFB assays provides operational flexibility for different testing settings. The fluorescence-based assay is more suitable for laboratory or instrument-assisted environments, where real-time signal monitoring, objective fluorescence measurement, and relatively higher-throughput testing can be performed. This format may be preferred when more standardized result interpretation, amplification curve monitoring, or semi-quantitative assessment is required. In contrast, the LFB-based assay is more suitable for settings with limited equipment, such as primary-care facilities, field screening, or resource-limited laboratories. The LFB format enables naked-eye interpretation without specialized instruments, making it useful for rapid preliminary screening and decentralized testing. Of note, visual interpretation may be affected by weak bands or operator subjectivity. Therefore, the fluorescence format may be preferable when instrumentation is available and more objective readout is needed, whereas the LFB format may be preferable when simplicity, portability, and equipment-free detection are prioritized.

Simulated clinical samples were included in this study to preliminarily evaluate the performance of the newly developed assays in a sample background closer to respiratory specimens. Given the unavailability of real RSV-positive clinical samples during the study period, the use of simulated samples provided an initial assessment of whether the RSV-RPA-FLU and RSV-RPA-LFB assays could maintain detectable performance in a clinically relevant matrix rather than only in purified plasmid systems. The results support the analytical feasibility of these assays and suggest their potential for future application in RSV detection from respiratory specimens.

Moreover, although the present study focused on the detection and differentiation of RSV-A and RSV-B, the dual-readout RPA strategy has potential extensibility to other pathogens. By selecting conserved and pathogen-specific genomic regions and redesigning corresponding primers and probes, this platform could be adapted for rapid nucleic acid detection of additional respiratory pathogens or other infectious agents. The availability of both fluorescence and LFB readout formats may further support its use in different testing environments. Nevertheless, such extension would require target-specific assay optimization and systematic validation, including analytical sensitivity, specificity, cross-reactivity, and clinical performance evaluation.

There are still some limitations in this study. First, the current specificity evaluation panel only included 11 non-RSV pathogens due to the lack more available nucleic acid samples. Future studies should include more closely related and clinically co-circulating respiratory viruses to further confirm the specificity and robustness of the assays. Second, the lack of validation using real clinical specimens limits the strength of evidence supporting the clinical applicability of the assay. Future studies should include well-characterized RSV-positive clinical samples from diverse geographic regions, age groups, and disease severities to further evaluate the diagnostic performance and generalizability of the assay.

In conclusion, the RSV-RPA-FLU assay and RSV-RPA-LFB assay developed in this study showed favorable analytical sensitivity and specificity, and operational simplicity for the detection and differentiation of RSV-A and RSV-B. These features indicate that the assays have potential as rapid molecular detection platforms, particularly for use in settings requiring simple and timely RSV screening.

## Data Availability

The original contributions presented in the study are included in the article/**Supplementary Material**. Further inquiries can be directed to the corresponding authors.
