# Supplementary material for: Rapid, sensitive, and highly specific diagnosis of respiratory syncytial virus using recombinase polymerase amplification-based biosensor and fluorescence detection
Source: Front Cell Infect Microbiol. 2026 Jul 1;16:1838582. doi: 10.3389/fcimb.2026.1838582 (PMC13368488; doi:10.3389/fcimb.2026.1838582)
Supplement: Supplementary file 1 [file Table1.docx]

**Supplementary Materials**

**Supplementary tables**

Table S1 Oligonucleotide sequences used in this study

| Sequence name | Sequence (5'-3') | Length (bp/nt) | GC content (%) | hairpin ΔG (kcal/mole) | self-dimer ΔG (kcal/mole) | BLAST identity to target (%) | Query coverage to target (%) |
| --- | --- | --- | --- | --- | --- | --- | --- |
| Forward primer (A) | AAATTAAGTGAAATACTAGGAATGCTTCACAC | 32 | 31.2 | -3.08 | -6.82 | 100 | 100 |
| Reverse primer(A) | TAGATCATTGTCACTATCATTCCCTTCCAATA | 32 | 34.4 | -0.38 | -5.37 | 100 | 100 |
| Reverse primer (A)* | Biotin-TAGATCATTGTCACTATCATTCCCTTCCAATA | 32 | 34.4 | -0.38 | -5.37 | 100 | 100 |
| Forward primer(B) | ATTAATGACCAATGATAGGTTAGAGGCTATG | 31 | 35.5 | -0.41 | -7.8 | 100 | 100 |
| Reverse primer(B) | GTTGATTCAATGGATTGATGTCTGTTTTATTG | 32 | 31.2 | -1.26 | -6.95 | 100 | 100 |
| Reverse primer(B)* | Biotin-GTTGATTCAATGGATTGATGTCTGTTTTATTG | 32 | 31.2 | -1.26 | -6.95 | 100 | 100 |
| exo probe (A) | GAAATGATAGAAAAAATCAGAACTGAAGCA(dT-FAM)(THF)(dT-BHQ1)AATGACCAATGACAGATTAG-C3 | 52 | 30.8 | -1.77 | -11.71 | 100 | 100 |
| exo probe (B) | CTTAATCCAACTTCCAAAAAATTGAGTGAC(dT-VIC)(THF)(dT-BHQ-1)GTTGGAAGACAACGA-C3 | 47 | 36.2 | -5.22 | -12.09 | 100 | 100 |
| nfo probe (A) | FAM-GAAATGATAGAAAAAATCAGAACTGAAGCAT(THF)TAATGACCAATGACAGATTAG-C3 | 52 | 30.8 | -1.77 | -11.71 | 100 | 100 |
| nfo probe (B) | DIG-CTTAATCCAACTTCCAAAAAATTGAGTGACT(THF)TGTTGGAAGACAACGA-C3 | 47 | 36.2 | -5.22 | -12.09 | 100 | 100 |

A, primers for RSV-A; B, primers for RSV-B; *, primers labeled with biotin for LFB-based assay。 Sequence length, GC content, hairpin ΔG, self-dimer ΔG, BLAST identity, and query coverage were evaluated for each primer and probe. GC content, hairpin ΔG, and self-dimer ΔG values were calculated using IDT OligoAnalyzer. For hairpin and self-dimer analyses, the most negative predicted ΔG value was recorded. The following reference criteria were used for interpretation: GC content of 30–70%, hairpin ΔG preferably ≥ −5 kcal·mol⁻¹, self-dimer ΔG acceptable ≥ −6 kcal·mol⁻¹, and 100% BLAST identity and 100% query coverage to the intended target sequence. More negative self-dimer ΔG values were considered to indicate potential oligonucleotide self-interaction in silico and were further evaluated by experimental validation. Primer/probe suitability was determined based on both in silico analysis and experimental performance.

Table S2 Pathogens used in this study for analytical specificity analysis

| Pathogen | Source of Strain* | RSV-RPA-FLU# | RSV-RPA-LFB# |
| --- | --- | --- | --- |
| Influenza A Virus | CDC | N | N |
| Influenza B Virus | CDC | N | N |
| Parainfluenza Virus 1 | CDC | N | N |
| Parainfluenza Virus 3 | CDC | N | N |
| Human Rhinovirus | CDC | N | N |
| Adenovirus | CDC | N | N |
| Epstein-Barr Virus | CDC | N | N |
| Rubella Virus | CDC | N | N |
| Measles Virus | CDC | N | N |
| Sendai Virus | CDC | N | N |
| Dengue Virus | CDC | N | N |

*CDC, Chinese Center for Disease Control and Prevention.

#, N, negative result.

Table S3 Optimized reaction systems for RSV-RPA-FLU and RSV-RPA-LFB assays

|  | RSV-RPA-FLU | RSV-RPA-LFB |
| --- | --- | --- |
| Forward primer (A) | 1.05 μl | 0.1 μl |
| Reverse primer (A) | 1.05 μl |  |
| Reverse primer (A)* |  | 0.1 μl |
| Forward primer (B) | 1.1 μl | 0.9 μl |
| Reverse primer (B) | 1.1 μl |  |
| Reverse primer (B)* |  | 0.9 μl |
| Probe(A)-FLU | 0.5 μl |  |
| Probe(B)-FLU | 0.5 μl |  |
| Probe(A)-LFB |  | 0.1 μl |
| Probe(B)-LFB |  | 0.9 μl |
| Primer free rehydration buffer | 29.5 μl | 29.5 μl |
| Distilled water | 10.7 μl | 13 μl |
| Template | 2 μl | 2 μl |
| Magnesium acetate | 2.5 μl | 2.5 μl |

A, primers for RSV-A; B, primers for RSV-B; *, primers labeled with biotin for LFB-based assay. Concentration of primers and probes was 10 μM, and the magnesium acetate was 280 mM.

Table S4 Confirmation of the putative lowest detectable concentrations using 20 independent replicate reactions.

| Assay | Subtype | Tested concentration | Positive / total replicates | Positivity rate | Wilson 95% CI | Confirmation criterion |
| --- | --- | --- | --- | --- | --- | --- |
| RSV-RPA-FLU | RSV-A | 6.05×10^4^ copies/μL | 20/20 | 100% | 0.839–1.000 | Met |
|  | RSV-B | 7.80×10^4^ copies/μL | 19/20 | 95% | 0.764–0.991 | Met |
| RSV-RPA-LFB | RSV-A | 6.05×10^3^ copies/μL | 20/20 | 100% | 0.839–1.000 | Met |
|  | RSV-B | 7.80×10^2^ copies/μL | 19/20 | 95% | 0.764–0.991 | Met |

Table S5 Comparison of the existing methods with the newly developed method in this study

| Method | Sensitivity | Assay time | Equipment requirement | Cost | Main advantages | Main limitations |
| --- | --- | --- | --- | --- | --- | --- |
| RT-qPCR | High | 1–2 h | Real-time PCR instrument | Relatively high | High sensitivity and specificity; widely used as reference method | Requires specialized equipment and trained personnel |
| Antigen test | Moderate to low | 10–30 min | Minimal | Low | Rapid, simple, suitable for preliminary screening | Lower sensitivity, especially in samples with low viral load |
| RPA-CRISPR | High | 30–60 min | Incubator/fluorescence reader or lateral flow strip | Moderate to high | High sensitivity and specificity; suitable for molecular detection | Additional CRISPR reaction components and workflow complexity |
| RSV-RPA-FLU | High analytical sensitivity | Approximately 40 min | Simple fluorescence reader | Moderate/low | Rapid, sensitive, closed-tube fluorescence readout | Clinical performance requires further validation |
| RSV-RPA-LFB | High analytical sensitivity | Approximately 40 min | Lateral flow strip; no complex instrument | Moderate/low | Visual detection, portable, suitable for low-resource settings | Clinical performance requires further validation |

Supplementary Figures

**
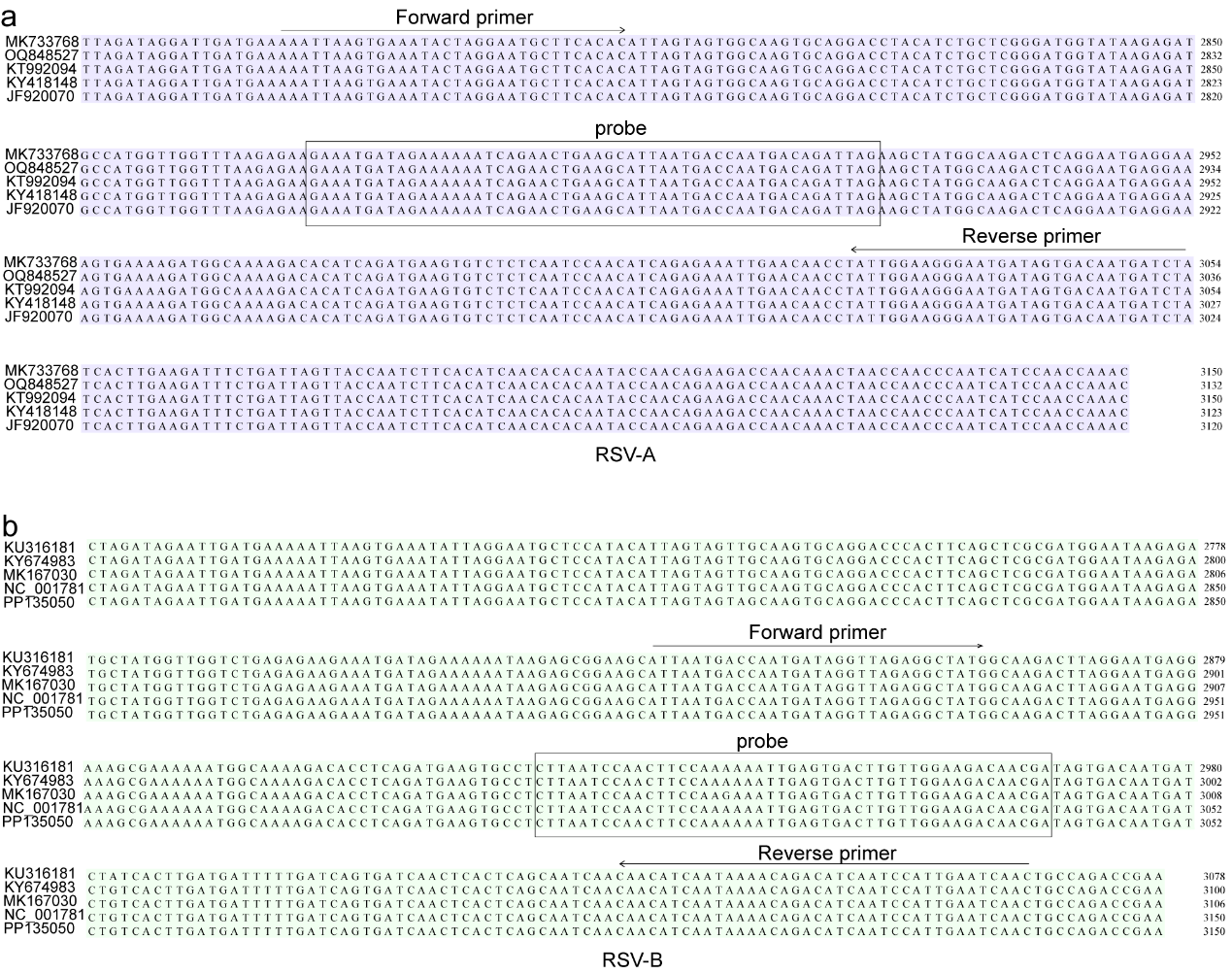
Fig. S1 Sequence alignment of RSV-A and RSV-B**

(a) Multiple sequences alignment of RSV-A ; (b) Multiple sequences alignment of RSV-B


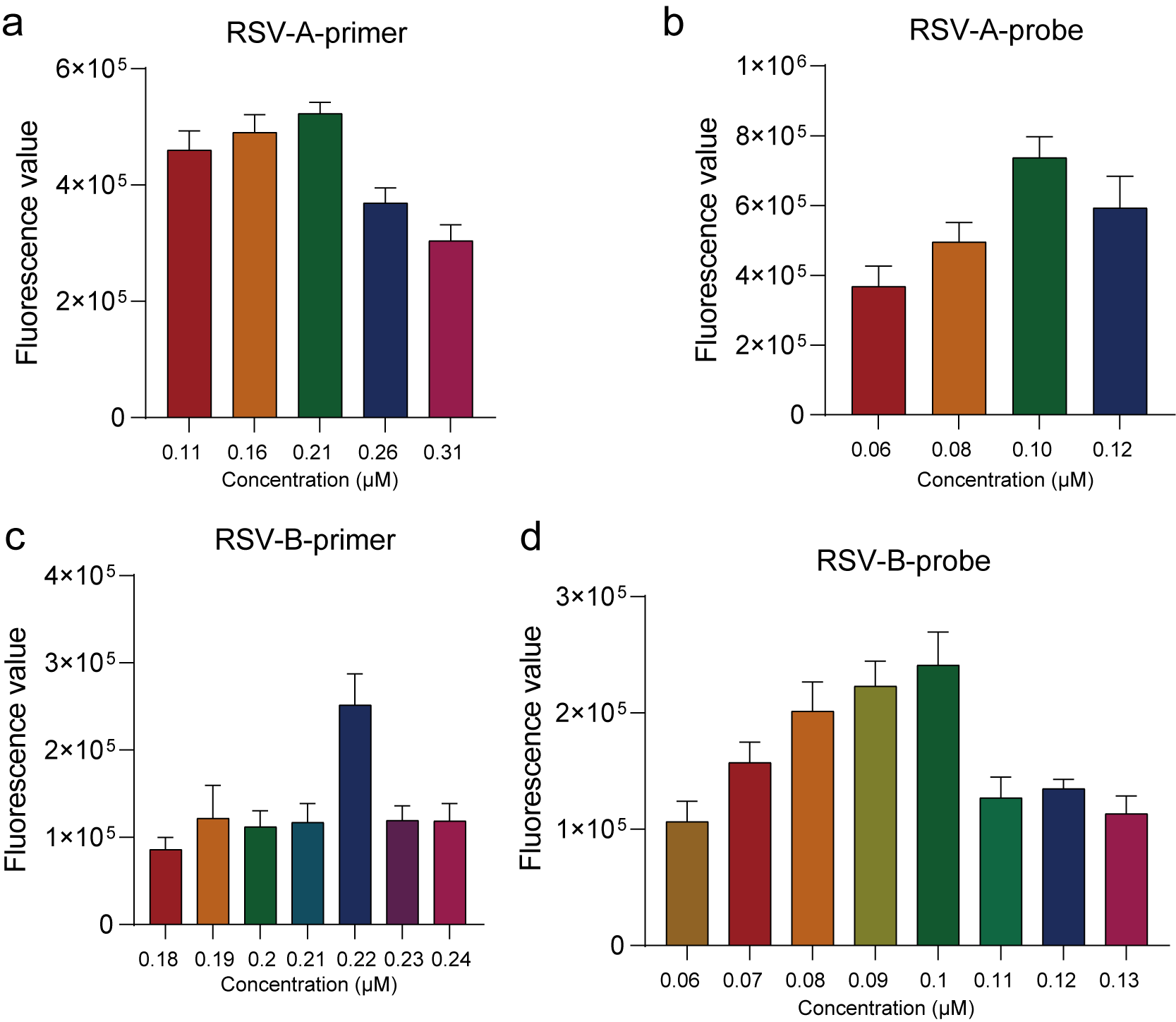


**Fig. S2 Primer and probe optimization**

RSV-A and RSV-B were used as detection targets. Single-factor variable analysis was applied to sequentially optimize primer and probe concentrations in the reaction system. (a) RSV-A primer concentration screening with gradients from 0.11 μM to 0.31 μM. Optimal concentration: 0.21 μM. (b) RSV-A probe concentration screening with gradients from 0.06 μM to 0.12 μM. Optimal concentration: 0.1 μM. (c) RSV-B primer concentration screening with gradients from 0.18 μM to 0.24 μM. Optimal concentration: 0.22 μM. (d) RSV-B probe concentration screening with gradients from 0.06 μM to 0.13 μM. Optimal concentration: 0.1 μM.

**
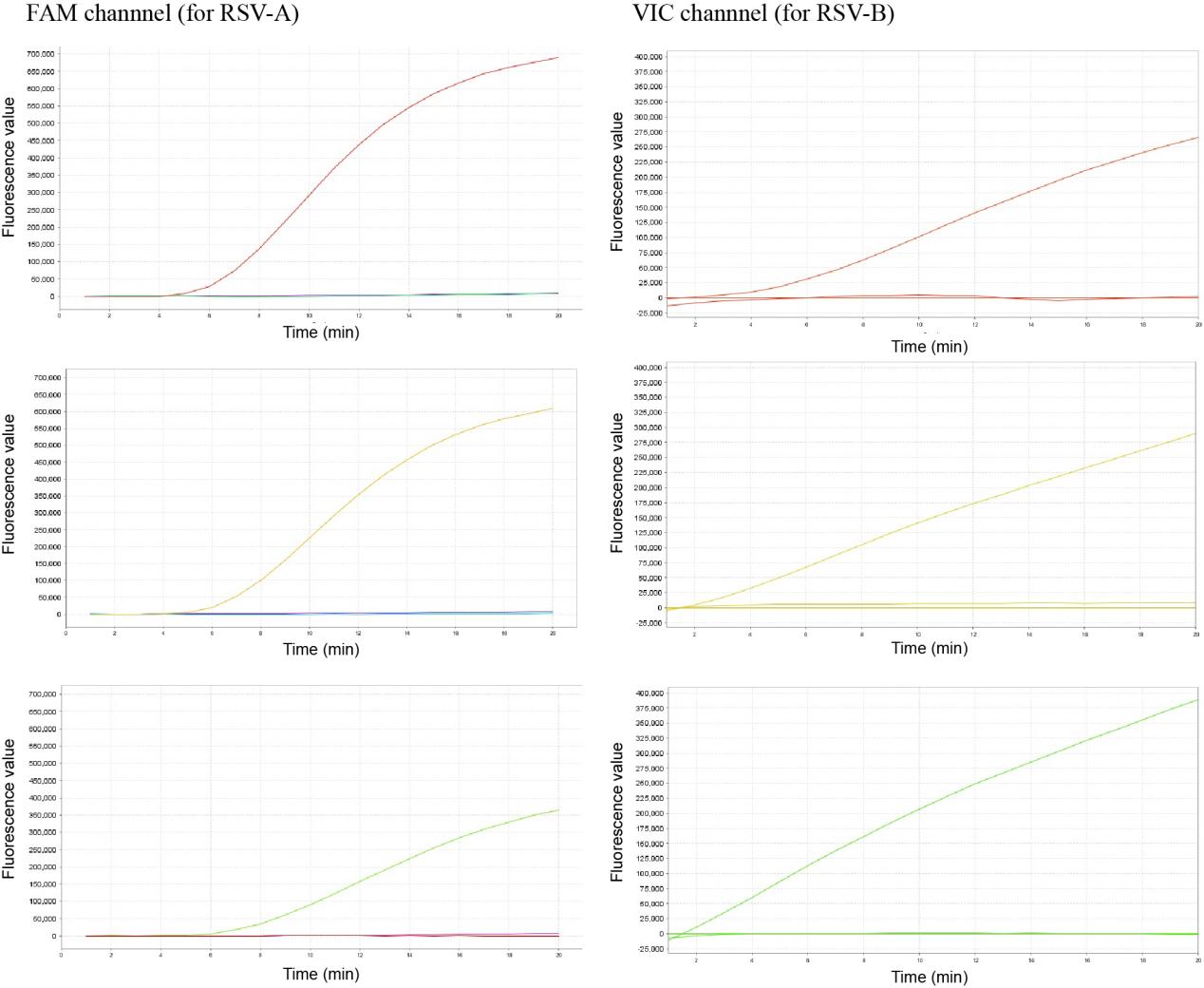
Fig. S3 Raw instrument-output fluorescence plots for confirmation of RSV-RPA-FLU assay**

The plots were exported directly from the instrument/software without smoothing, normalization, or graphical optimization. Each condition was tested in triplicate.

**
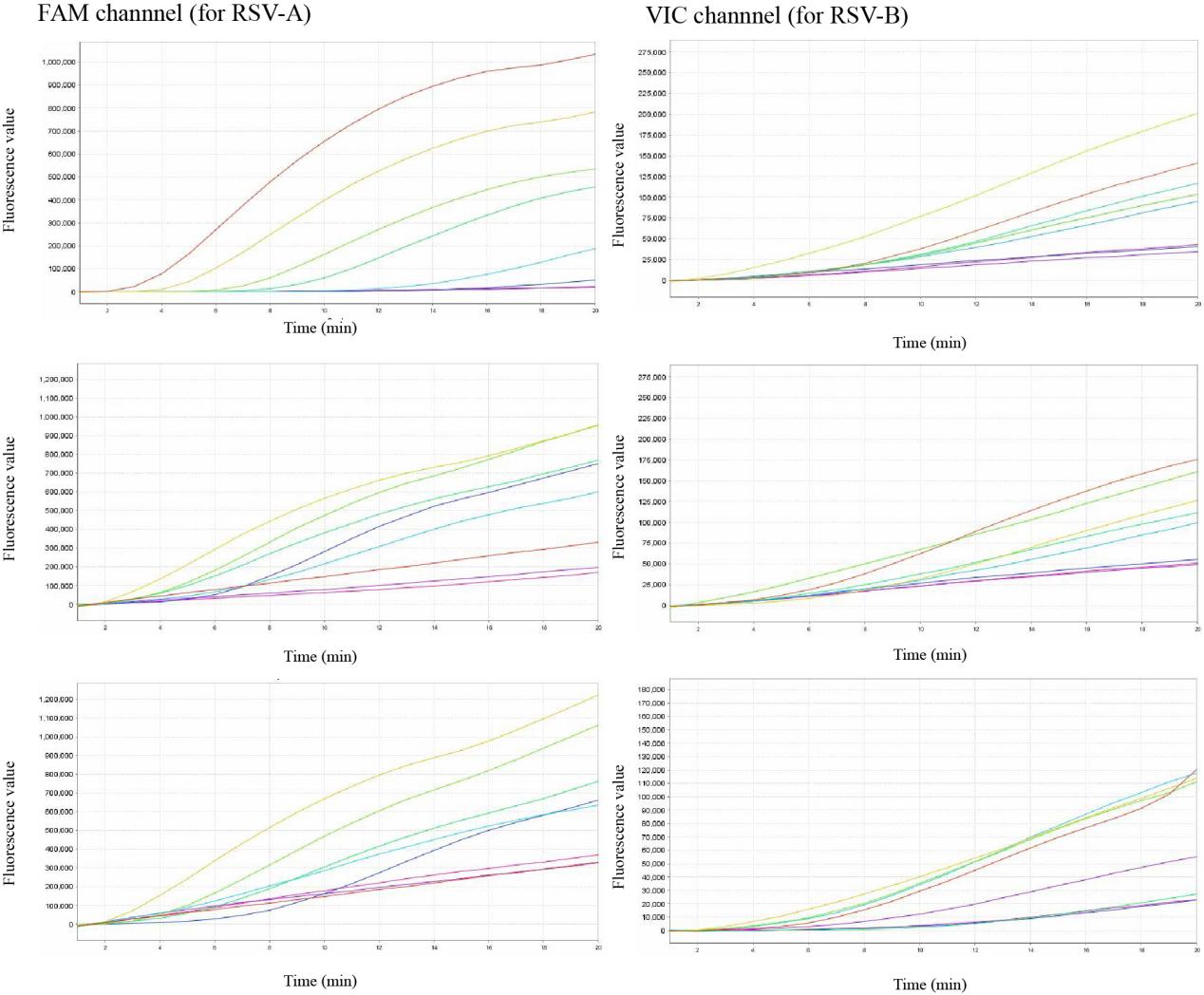
Fig. S4 Raw instrument-output fluorescence plots for analytical sensitivity of RSV-RPA-FLU assay**

The plots were exported directly from the instrument/software without smoothing, normalization, or graphical optimization. Each condition was tested in triplicate.

**
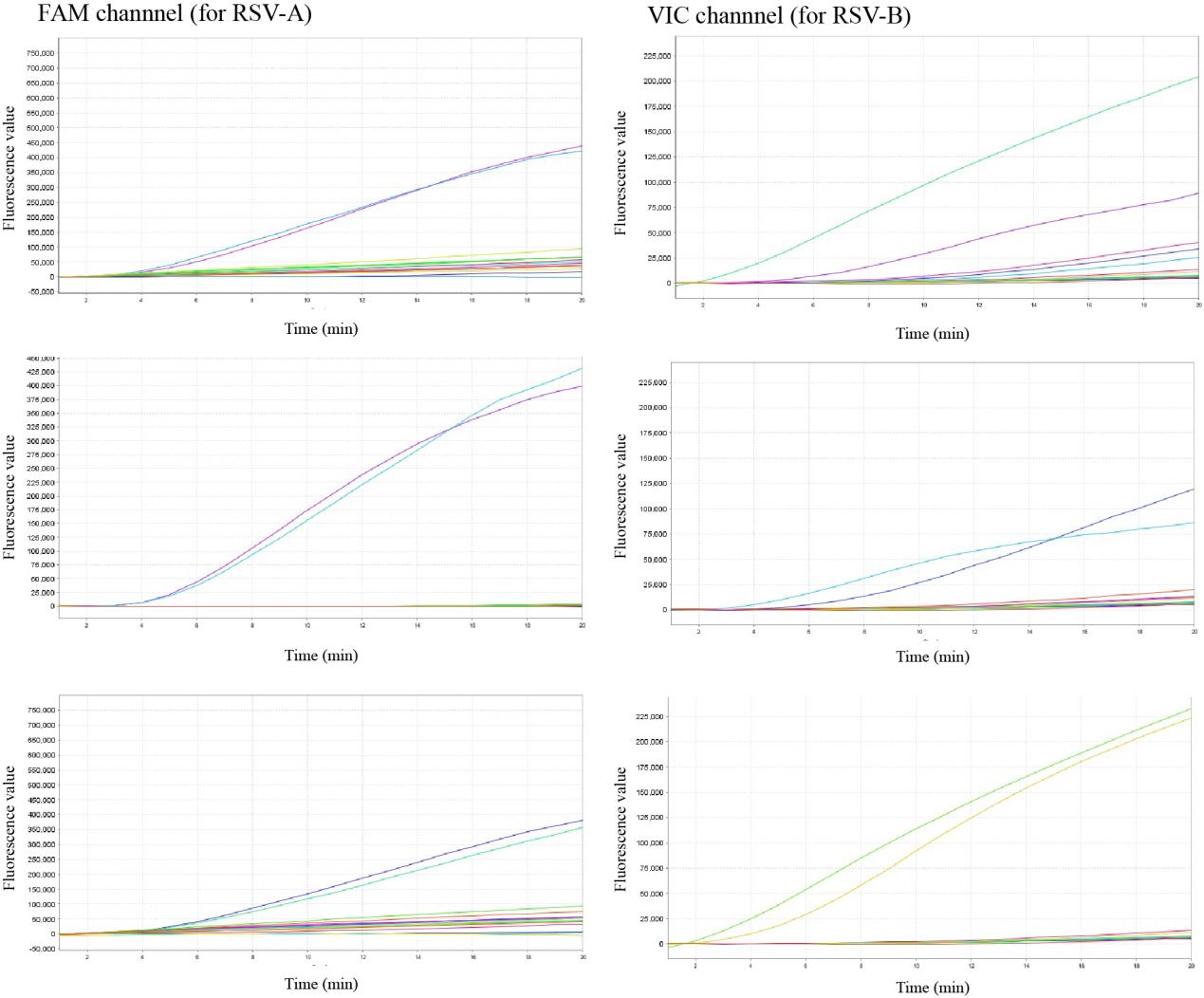
Fig. S5 Raw instrument-output fluorescence plots for analytical specificity of RSV-RPA-FLU assay**

The plots were exported directly from the instrument/software without smoothing, normalization, or graphical optimization. Each condition was tested in triplicate.

**
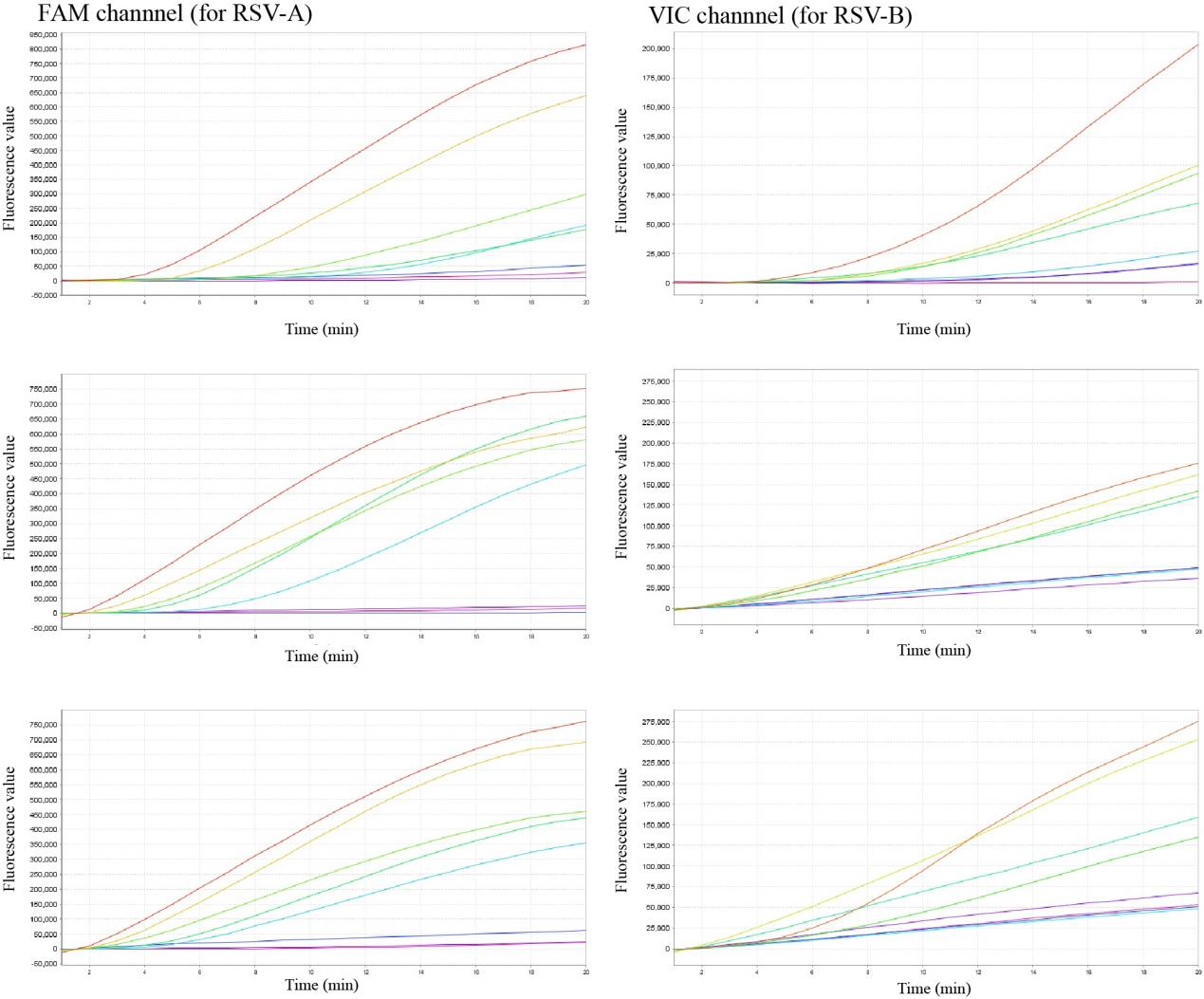
Fig. S6 Raw instrument-output fluorescence plots for preliminary evaluation of the RSV-RPA-FLU assay in plasmid-spiked nasopharyngeal swab matrix**

The plots were exported directly from the instrument/software without smoothing, normalization, or graphical optimization. Each condition was tested in triplicate.
